# Genetics of Aggression in Alzheimer’s Disease (AD)

**DOI:** 10.3389/fnagi.2017.00087

**Published:** 2017-04-10

**Authors:** Walter J. Lukiw, Evgeny I. Rogaev

**Affiliations:** ^1^Louisiana State University (LSU) Neuroscience Center, Louisiana State University Health Science Center New Orleans, LA, USA; ^2^Department of Ophthalmology, Louisiana State University Health Science Center New Orleans, LA, USA; ^3^Department of Neurology, Louisiana State University Health Science Center New Orleans, LA, USA; ^4^Bollinger Professor of Alzheimer’s disease (AD), Louisiana State University Health Sciences Center New Orleans, LA, USA; ^5^Vavilov Institute of General Genetics, Russian Academy of Sciences Moscow, Russia; ^6^Center for Brain Neurobiology and Neurogenetics, Institute of Cytology and Genetics, Siberian Branch of the Russian Academy of Sciences Novosibirsk, Russia; ^7^Department of Psychiatry, Brudnick Neuropsychiatric Research Institute, University of Massachusetts Medical School Worcester, MA, USA; ^8^School of Bioengineering and Bioinformatics, Lomonosov Moscow State University Moscow, Russia

**Keywords:** aggression-Alzheimer’s disease (AD), retrogenesis and schizophrenia, androgen receptor (AR), brain derived neurotrophic factor (BDNF), tryptophan hydroxylase (TPH), neuronal specific nitric oxide synthase (NOS1), catechol-O-methyl transferase (COMT), dopamine beta-hydroxylase (DBH)

## Abstract

Alzheimer’s disease (AD) is a terminal, age-related neurological syndrome exhibiting progressive cognitive and memory decline, however AD patients in addition exhibit ancillary neuropsychiatric symptoms (NPSs) and these include aggression. In this communication we provide recent evidence for the mis-regulation of a small family of genes expressed in the human hippocampus that appear to be significantly involved in expression patterns common to both AD and aggression. DNA array- and mRNA transcriptome-based gene expression analysis and candidate gene association and/or genome-wide association studies (CGAS, GWAS) of aggressive attributes in humans have revealed a surprisingly small subset of six brain genes that are also strongly associated with altered gene expression patterns in AD. These genes encoded on five different chromosomes (chr) include the androgen receptor (AR; chrXq12), brain-derived neurotrophic factor (BDNF; chr11p14.1), catechol-O-methyl transferase (COMT; chr22q11.21), neuronal specific nitric oxide synthase (NOS1; chr12q24.22), dopamine beta-hydroxylase (DBH chr9q34.2) and tryptophan hydroxylase (TPH1, chr11p15.1 and TPH2, chr12q21.1). Interestingly, (i) the expression of three of these six genes (COMT, DBH, NOS1) are highly variable; (ii) three of these six genes (COMT, DBH, TPH1) are involved in DA or serotonin metabolism, biosynthesis and/or neurotransmission; and (iii) five of these six genes (AR, BDNF, COMT, DBH, NOS1) have been implicated in the development, onset and/or propagation of schizophrenia. The magnitude of the expression of genes implicated in aggressive behavior appears to be more pronounced in the later stages of AD when compared to MCI. These recent genetic data further indicate that the extent of cognitive impairment may have some bearing on the degree of aggression which accompanies the AD phenotype.

## Introduction

There are currently 5.4 million Americans afflicted with Alzheimer’s disease (AD) and one in nine Americans (11.1%) over the age of 65 have AD^1^. In the later stages, about 45% of all AD patients exhibit hostility and aggression towards their fellow patients, caregivers and/or families (Borroni et al., 2010; Ballard and Corbett, 2013; Fernàndez-Castillo and Cormand, 2016; Liu et al., 2016; Macfarlane and O’Connor, 2016; Wharton et al., 2016). Aggressive behaviors in AD represent a significant public healthcare concern and a social and clinical challenge for institutionalized healthcare. The aggression associated with AD is expressed as an overt and/or often harmful social interaction with the primary intention of inflicting damage or other unpleasantness upon other individuals that may occur either without provocation or in retaliation. Aggressive behavior in AD: (i) can take a variety of neuropsychiatric (NPS) forms; (ii) may be communicated verbally or non-verbally; or (iii) may be expressed physically. There are often multiple forms of aggression in the AD patient including defensive (fear-induced) aggression, predatory aggression, dominance aggression, inter-male aggression, resident-intruder aggression, maternal aggression, sex-related aggression, territorial aggression, isolation-induced aggression and irritability-associated aggression (Fernàndez-Castillo and Cormand, 2016; Liu et al., 2016; Macfarlane and O’Connor, 2016; Wharton et al., 2016). Clinical and social aspects of these types of AD-associated aggression are the subject of several recent excellent reviews and include the most up-to-date pharmacological strategies and therapeutic advances to specifically address the aggressive AD phenotype (Ballard and Corbett, 2013; Fernàndez-Castillo and Cormand, 2016; Liu et al., 2016; Macfarlane and O’Connor, 2016; Wharton et al., 2016). This original research article provides new data on the overlap in the expression of brain-enriched genes in AD with those implicated in aggression and incorporates the findings of the most recent peer-reviewed research on emerging concepts of the genetics linked to aggression in AD.

## Materials and Methods

### Brain Sample Selection and Quality Control

Brain tissues were used in accordance with the institutional review board (IRB)/ethical guidelines at the Louisiana State University Health Sciences Center (LSUHSC) and the donor institutions, and CERAD/NIH criteria were used to categorize all AD tissues in accordance with established guidelines (Cui et al., 2005, 2010; Lukiw et al., 2008; Montine et al., 2016). We used mRNA expression profiles and DNA-array data from a study of 12 mild-cognitively impaired (MCI) subjects (*N* = 12) and their controls (*N* = 12), and 12 age-matched sporadic AD hippocampal CA1 (*N* = 12) and their controls (*N* = 12). DNA array data was performed using state-of-the-art microfluidics hybridization platforms through an ongoing collaboration with Drs. Cristoff Eicken, Chris Hebel, Jason Mulcahey and Kyle Navel at LC Sciences (Houston, TX, USA); mRNA expression profiles and trends were independently corroborated and verified using RT-PCR or Northern blotting in previously published work from this lab (Cui et al., 2005, 2010; Lukiw et al., 2008; Bhattacharjee et al., 2016). Both the MCI and AD groups had their own control groups because of the age-differences between MCI and AD and our understanding that age is the most significant risk factor for the development of AD (Table 1). All control, MCI and AD samples had the incidence of disease verified by post-mortem evaluation; there were no significant differences in age, gender, drug history, total RNA yield, RNA quality, RNA integrity numbers (RIN) amongst the three groups (see Table 1).

**Table 1:** **Summary of tissues used from each case group in this study**.

Case group	*N*	Age*^1^ x ± SD	Age*^1^ range	PMI*^2^ range	SP/NFT*^3^	RNA A_260/280_	RNA 28S/18S	RNA yield*^4^
**C1**	12	64.1 ± 8.1	64–78	2.5–3.5	1/3	2.07–2.15	1.45–1.55	1.15–1.35
**MCI**	12	64.8 ± 8.6	61–71	2.6–3.7	4/8	2.08–2.16	1.45–1.6	1.15–1.42
**C2**	12	71.5 ± 9.0	63–77	1.1–4.3	1/3	2.05–2.15	1.45–1.6	1.12–1.42
**AD**	12	72.1 ± 7.5	65–79	1.1–4.2	10/18	2.08–2.12	1.45–1.5	1.05–1.51

### Expressed Genes Involved in AD and Aggression

**Figure 1:**
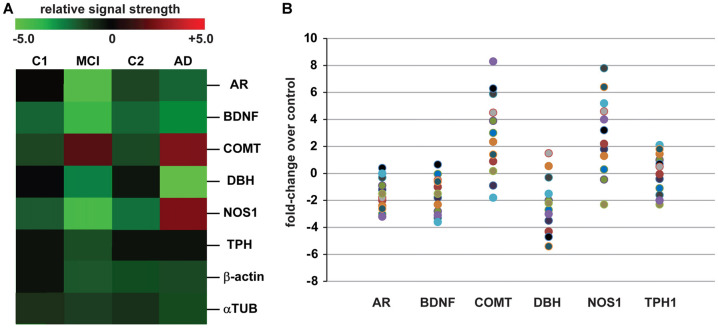
**Analysis of expressed genes common to both Alzheimer’s disease (AD) and aggression. (A)** mRNA transcriptomic arrays were used to quantify the levels of some of the most mis-regulated genes in mild-cognitive impairment (MCI) and AD brain samples compared to an MCI control **(C1)** and an independent AD control **(C2)**; these were compared to unchanging β-actin and/or α-tubulin (αTUB) levels in the same sample; gene expression patterns were next compared to those also mis-regulated in “*the aggressive phenotype*” as recently reported by Fernàndez-Castillo and Cormand (2016); expressed gene alterations common to both AD and aggression included the androgen receptor (AR), brain-derived neurotrophic factor (BDNF), catechol-O-methyl transferase (COMT), dopamine beta-hydroxylase (DBH), nitric oxide synthase (NOS1) and tryptophan hydroxylase (TPH1 shown); genes including COMT and NOS1, and TPH1 to a lesser extent, exhibit increased expression in the transition from MCI to AD; for example TPH1 showed a modest 0.2-fold increase in expression AD over MCI while NOS1 and COMT displayed 2.8- and 2.9-fold increases in expression (*p* < 0.05, ANOVA); data for C1, C2, MCI and AD *N* = 12 cases each; the magnitude of expression for each AD case is quantified in **(B)** for all 12 samples studied; great care was taken in the selection of control and age-matched AD samples; all brain samples were from the hippocampal CA1; there were no significant differences in age, age range, gender, post-mortem interval (PMI) range, or RNA quality between the two groups **(Table 1)**; in **(B)** data for AR, BDNF, COMT, DBH, NOS1 and TPH1 are compared to controls for each sample **(Table 1)**; interestingly, genes such as COMT, BDNF and NOS1: (i) exhibit a trend for wider variation in their expression from sample to sample, and this is in agreement with reports of both up-and down-regulation of their abundance in the literature (see above); and (ii) COMT and NOS1 display the largest general trend for up-regulation in AD vs. control **(C2)**; DBH exhibits a general trend for down-regulation **(A)**. Genechip data for **(B)** is given in Supplementary data in Table S1. Other relevant DNA array data for these and other genes expressed in AD and age-matched controls from our laboratories have been previously published (Colangelo et al., 2002; Cui et al., 2005; Lukiw et al., 2008). Ongoing future studies will benefit from larger sample sizes, more homogeneous phenotypes and standardized measurements to identify expressed genes that underlie aggressive behaviors which accompany the AD process.

The data derived from our DNA/mRNA transcriptomic array analysis were correlated and compared with those of a very recent article by Fernàndez-Castillo and Cormand (2016) who studied aggressive behavior in humans through the analysis of genes and pathways identified via candidate gene association and/or genome-wide association studies (CGAS, GWAS). Only genes of the highest significance, either up-or-down-regulated, were selected for further study, analysis and interpretation. Just six genes were found to most significantly overlap in the incidence of AD and aggression and these included the androgen receptor (AR; chrXq12), brain-derived neurotrophic factor (BDNF; chr11p14.1), catechol-O-methyl transferase (COMT; chr22q11.21), neuronal specific nitric oxide synthase (NOS1; chr12q24.22), dopamine beta-hydroxylase (DBH chr9q34.2) and tryptophan hydroxylase (TPH1, chr11p15.1 and TPH2, chr12q21.1; Figure 1).

## Results

Table 1 describes the case group (control for MCI **(C1)**, MCI, control for AD **(C2)** and AD), mean and 1 SD of age, post-mortem interval (PMI), senile plaque (SP) and neurofibrillary tangle (NFT) density and RNA quality control parameters and RNA yield for the samples analyzed. There were no significant differences in mean age, PMI, gender, RNA quality control or RNA yield between MCI or AD and their controls; MCI displayed 2.7–4 times the density of SP and NFT as its control **(C1)**; AD displayed 6.1–10 times the density of SP and NFT as its control **(C2)**; *p* < 0.05 (ANOVA); all samples were of the highest purity available from multiple sources of post-mortem brain human samples (Cui et al., 2005; Lukiw et al., 2008; Zhao et al., 2016). High quality RNA samples were subsequently analyzed using mRNA transcriptomic arrays employing μParaflo^®^ Microfluidic Biochip Technologies that interrogate the abundance and speciation of ~27,000 human mRNAs (LC Sciences, Houston, TX, USA); digitized data were presented in the form of cluster diagrams (Cui et al., 2005; Lukiw et al., 2008); Figure 1A describes the results in a color-coded cluster diagram of the most changed genes in MCI or AD vs. controls for an overlapping family of brain genes involved in aggressive behaviors (Fernàndez-Castillo and Cormand, 2016). These genes include AR, BDNF, COMT, DBH, NOS1and TPH1 (TPH2 was not analyzed in these assays). Individual AD signal yield expressed as fold-change over controls are described in a scatter plot in Figure 1B. Individual values >0 indicate up-regulation of expression and values <0 indicate decreased expression. Interestingly, the AR, BDNF and TPH1 gene expression patterns all displayed a smaller general variability vs. a wider variability in the expression of genes for COMT, DBH and NOS1 in the AD brain (Figure 1B). This may explain, for example, reports on both up- and down-regulated expression of COMT, DBH and NOS1 in the AD brain (see below). The following is a brief discussion of these six genes and their relevance to aggression and AD, highlighting recent advances, particularly within the last 10 months, in this understudied research area.

## Discussion

### Androgen Receptor (AR, ANDR; chrXq12)

Circulating sex steroidal hormones such as testosterone that play overlapping roles in cognition and aggression have been widely implicated in the etiopathology of AD. While the AR is abundantly expressed in both sexes, in AD expression of the AR has been found to be generally down-regulated, especially in aging males (Butchart et al., 2013; Dart et al., 2013; Fedotova et al., 2016; Jia et al., 2016). Interestingly the steroid-hormone testosterone-activated transcription factor-signaling AR (ANDR) is known to exert regulatory effects on synaptic plasticity and improve cognitive deficits in AD patients and transgenic rodent models for AD (TgAD) but the underlying mechanisms of androgenic action on cognitive performance remain unclear. Testosterone, via the AR, increases synaptophysin expression and improves synaptic plasticity and cognitive metrics in both Aβ42 peptide-hippocampal injected male rats and in the senescence-accelerated mouse prone 8 (SAMP8) TgAD murine model (Huo et al., 2016; Jia et al., 2016). In a recent double-blind, placebo-controlled, between-subject designed study, exogenous administration of testosterone to healthy adult men potentiated the AR-enriched hippocampus, amygdala and hypothalamus—anatomical regions known to be involved in the stimulation of aggressive behavior (Carré et al., 2016). Several studies have further suggested that new treatments targeted toward preventing synaptic pathology in AD or in TgAD models may involve the use of androgen agonists and/or AR-acting drugs (Jia et al., 2016; Pan et al., 2016). Interestingly, polyprenol bioregulators such as *Ropren* (already used in AD treatment in Russia and Australia) and polyprenol 2,3-dihydro derivatives known as “*dolichols*” in gonadectomized rodents exhibit decreased 5-hydroxyindoleacetic acid (5-HIAA)/5-HT ratios in the hippocampus compared with DA and serotonin (5-HT) levels, and are associated with increased anxiolytic (antianxiety) behaviors in treated animals (Sugden et al., 2009; Fedotova et al., 2016). Therefore it may be able to design useful drugs that are able to negate aggression while promoting other useful aspects of testosterone and AR signaling including improvement in cognitive function.

### Brain-Derived Neurotrophic Factor (BDNF; chr11p14.1)

BDNF is the most widely distributed neurotrophin in the human CNS and plays several pivotal roles in neural development, survival, synaptic plasticity and cognition. This 247 amino acid neurotrophin participates in axonal growth, pathfinding and in the modulation of dendritic growth and morphology, is a master regulator of synaptic plasticity and thus plays a key role in memory formation and storage (Faria et al., 2014; Nagata et al., 2014, 2016; Song et al., 2015). Therefore, the involvement of BDNF in dementia has been extensively investigated, and several reports indicate that BDNF expression is decreased in hippocampus and neocortex of AD brains (Figure 1; Peng et al., 2005; Song et al., 2015). Interestingly, children with severe autism and aggression over-express the AD-relevant beta-amyloid precursor protein (βAPP) at two or more times the levels of children without autism and up to four times more than children with mild autism; βAPP-derived Aβ peptides can directly inhibit the proteolytic conversion of BDNF from pro-BDNF thus reducing its levels (Sokol et al., 2006; Tanila, 2017). Aβ peptides indirectly affect BDNF signaling at synapses by interfering with its axonal transport in part via interaction with the TrkB receptor (Durany et al., 2000; Tanila, 2017). That BDNF expression is associated with multiple independently-regulated promoters suggest that this unique genomic structure may provide flexibility to regulate BDNF signaling in distinct cell types and circuits that serve distinct molecular functions some of which may be involved in aggressive behaviors (Spalletta et al., 2010; Faria et al., 2014; Maynard et al., 2016). It is interesting to speculate that episodic or oscillatory forms of BDNF expression in AD may correlate with aggressive outbreaks or contribute to them via complex neurotrophic signaling pathways that are known to impact behavioral phenotype.

### Catechol-O-Methyl Transferase (COMT; chr22q11.21)

COMT catalyzes the transfer of a methyl group from S-adenosylmethionine to catecholamines, including the neurotransmitters DA, epinephrine and norepinephrine, resulting in one of the major degradative pathways of the catecholamine transmitters. COMT appears to play a prominent role in AD pathophysiology by affecting the metabolism of catecholamine neurotransmitters such as DA in the prefrontal cortex which are involved in working memory and executive functioning (Serretti and Olgiati, 2012). Similarly, COMT and DBH are directly involved with DA metabolism; this neurotransmitter system appears to be specifically targeted by the AD process (Martorana and Koch, 2014). While levels of COMT in sporadic AD appear to be highly variable, either up- or down-regulated, meta-analysis studies have identified a possible association between AD and the COMT Val158Met polymorphism in Asian but not in European populations (Lee and Song, 2014; Sauerzopf et al., 2017). More recent and comprehensive meta-analysis involving a review of 10 independent studies, which contained a total of 2777 AD cases and 2829 controls indicated that the COMT Val158Met polymorphism is associated with a decreased risk of AD in the Asian population, but not in the Caucasian or overall populations (Yan W. et al., 2016). Interestingly, genetic risk score based on the accumulation of multiple risk alleles in BDNF, COMT and APOE for AD combining multiple genetic influences may be more useful in predicting late-life cognitive impairment than individual polymorphisms (Wollam et al., 2015). As for other neurotrophins and neurotransmitters such as NOS1, periodic fluctuations in COMT expression in AD may correlate with aggressive behaviors and contribute to “*behavioral dys-homeostasis*” via complex and highly interactive neurotrophic signaling pathways that contribute to NPS events (Perroud et al., 2010; Clelland et al., 2016). Interestingly, prefrontal and temporal lobe dysfunction and mutations in the COMT gene have been linked to agitation and aggression in patients with schizophrenia, and deficits in cognitive function predispose patients with AD to agitation (Sachs, 2006; Clelland et al., 2016; Yan P. et al., 2016; Yan W. et al., 2016).

### Dopamine β-Hydroxylase (DBH; chr9q34.2)

Dopamine beta-hydroxylase (DBH), an oxidoreductase belonging to the copper-requiring type II, ascorbate-dependent monooxygenase family and present in the synaptic vesicles of postganglionic sympathetic neurons exists in both soluble and membrane-bound forms, depending on the absence or presence, respectively, of a signal peptide (Herrmann et al., 2004; Godar and Bortolato, 2014; Ross et al., 2015; Godar et al., 2016). DBH converts DA to norepinephrine (Ross et al., 2015). Like COMT and NOS1, DBH exhibit wide inheritable inter-individual variability in AD that is under genetic control (Figure 1; Mustapic et al., 2013; Ross et al., 2015). Decreases in DBH activity found in the early stages of AD may be a reflection of loss of noradrenergic (NA) neurons, and the treatment of early AD patients with selective NA reuptake inhibitors may be indicated in early stages of AD to compensate for loss of NA activities (Mustapic et al., 2013). The highly complex and interactive nature of neurotrophins and neurotransmitters in aggressive behaviors are underscored by recent observations that serotonin and DA interactions in the prefrontal cortex, along with other biological factors such as norepinephrine and testosterone have been found to contribute to the aggressive phenotype, as has monoamine oxidase A (MAOA) that catalyzes the degradation of brain serotonin, norepinephrine and DA (Haavik et al., 2008; Godar et al., 2016).

### Neuronal Specific Nitric Oxide Synthase (NOS1; chr12q24.22)

NOS1, a family of NO synthases which synthesize NO from L-arginine is a pivotal mediator in neurotransmission, spatial learning and cognition and has been repeatedly implicated in the neurotoxicity associated with stroke and neurodegenerative disease (Wultsch et al., 2007; Freudenberg et al., 2015; Austin and Katusic, 2016; Miszczuk et al., 2016; Wang et al., 2016). Interestingly, NO is a gaseous neurotransmitter that has also been implicated in a wide range of pathological behaviors including aggression, anxiety, depression and cognitive functioning. NOS1 knockdown mice display a characteristic behavioral profile consisting of reduced anxiety and aggression and impaired cognitive metrics including learning and memory (Wultsch et al., 2007; Miszczuk et al., 2016; Zhou et al., 2016). Recent studies have shown that deficits of endothelial NOS1 in AD play an important role in the phosphorylation of the AD-related lesion tau in murine APP/PS1 TgAD models (Austin and Katusic, 2016) while increased NOS-1 activity and NO transmission in the hippocampus was evidenced to modulate aggression (Miszczuk et al., 2016; Zhou et al., 2016). Again, the seemingly multiphasic bioavailability of NOS1 like that for COMT provides an equally oscillatory potential for episodic and complex behaviors such as aggression against a background of the inflammatory neurodegeneration and disorganized thinking that in part characterize cognitive disruption in AD.

### Tryptophan Hydroxylase (TPH1, chr11p15.1; TPH2, chr 12q21.1)

TPH catalyzes the first and rate limiting step in the biosynthesis of the monoamine hormone, neurotransmitter and neuromodulator serotonin (3-(2-aminoethyl)-1H-indol-5-ol, 5-hydroxytryptamine; 5-HT). Remarkably, about 90% of the human body’s total serotonin (and hence TPH biosynthetic systems) are located in the neuroendocrine entero-chromaffin cells in the human gastrointestinal (GI) tract, where it is used to regulate intestinal movements and participate in GI-neural tract signaling; the other 10% is synthesized by serotonergic neurons of the CNS where it functions in the regulation of neurite outgrowth, somatic morphology, growth cone motility, synaptogenesis, and control of dendritic spine shape and density, and behaviorally anger, aggression, mood, body temperature, appetite, sleep, pain and cognition. Serotonin acts via a heterogenic receptor family that includes G protein-coupled receptors and ligand-gated ion channels (New et al., 1998; Craig et al., 2004; Shearer et al., 2016; Wirth et al., 2016). TPH is encoded by two separate enzymes: TPH1 produces serotonin in the pineal gland and entero-chromaffin cells, while TPH2 produces serotonin in the Raphe nuclei of the brain stem and myenteric plexus. Interestingly, serotonin is phylogenetically the oldest neurotransmission system present involved in cognition and memory in both vertebrate and invertebrate species, and pathological alterations in 5-HT metabolism and/or down-regulation of serotonergic signaling have been associated with various pathophysiological conditions in the CNS including amyloidogenesis and SP formation, hyper-phosphorylation of tau and NFT formation, two classical aggregates that in part characterize AD brain neuropathology (McClam et al., 2015; Panza et al., 2015; Butzlaff and Ponimaskin, 2016; Wirth et al., 2016). Due to the extensive serotonergic denervation observed in AD and the important roles played by serotonin in both behavior and cognition, this neurotransmitter system has become a focus of dedicated research efforts to identify novel pathways in Aβ peptide or NFT assembly and/or aggregation (Butzlaff and Ponimaskin, 2016; Schneider et al., 2016; Wirth et al., 2016). Serotonin has been found to increase by 4-fold, respectively in AD raphe cell bodies over controls, while in amygdala synaptic terminals 5-HT was decreased to 0.4-fold in the same AD cases; the accumulation of TPH and its products in the raphe perikarya in AD as the result of diminished transport of TPH to axon terminals and the accumulation of oxidative metabolites of serotonin may contribute to the degeneration of serotonergic neurons in AD (Burke et al., 1990; Wirth et al., 2016). Recent studies have also shown that the administration of selective serotonin reuptake inhibitors (SSRI) to Tg-AD murine models both reduces the production of toxic Aβ peptides and SP formation (Sheline et al., 2014; Butzlaff and Ponimaskin, 2016). For example, SSRI such as the antianxiety/ antidepressant citalopram hydrobromide (Celexa) has been used in AD with varying success while antipsychotics remain the pharmacological agents with most evidence to support their use (Gallagher and Herrmann, 2015). On the other hand other recent investigations, as part of the recent Citalopram for Agitation in Alzheimer Disease (CitAD) study, indicate that higher doses of SSRIs such as citalopram actually may trigger aggression especially in patients with more severe cognitive impairment encountered in the more advanced stages of AD (Schneider et al., 2016).

### Genetics of Aggression in Schizophrenia

With a median incidence of 152 cases/M (million) persons (range 77–430 cases/M; c.i. 80%), schizophrenia is a relatively common and perplexing neurological disorder characterized by a series of NPSs that include abnormal psychosocial behavior, anxiety, disorganized thinking and impaired cognition, reduced social engagement and emotional expression, lack of motivation and a significantly increased incidence of aggression (McGrath et al., 2008; Soyka, 2011; Morris et al., 2015; Agarwal et al., 2016; Cocchi et al., 2016; Janoutová et al., 2016; Nowak et al., 2016). Globally, there are an estimated ~25 million cases of schizophrenia; males are more often affected than females (at a ratio of 1.4 to 1), environmental and lifestyle risk factors include birth in a winter month, at a high latitude, and in an urban setting, and societal problems, such as long-term unemployment, poverty, and homelessness are common (Agarwal et al., 2016; Janoutová et al., 2016; Nowak et al., 2016; van de Leemput et al., 2016). The average life expectancy of people with schizophrenia is ~10–25 years less than the average, and in the US an estimated 16,000 people die from behaviors related to, or caused by, schizophrenia each year (Agarwal et al., 2016; Janoutová et al., 2016; Nowak et al., 2016; van de Leemput et al., 2016). As with AD, aggressive acts committed by patients with schizophrenia are a major public health concern affecting patients, their families and caregivers as well as organized healthcare and the criminal-justice system. Also similar to AD, schizophrenia appears to target high-level processing functions associated with the neocortex and cortical synaptic connectivity; the genetic contributions to schizophrenia are complex and multifactorial with the integrated contributions of brain genes thought to be centrally involved in auditory processing, altered mood and behavior, anxiety and disorganized thought (Coyle et al., 2016). As previously mentioned, five of these six genes (AR, BDNF, COMT, DBH, NOS1) identified to be dysregulated in this study in AD and aggression have been implicated in the development, onset and/or propagation of schizophrenia. Interestingly both “*dopaminergic and serotoninergic hypotheses for schizophrenia*”, involving an elevated genetically-based capacity for striatal increased L-phenylalanine- and/or L-tyrosine-derived DA biosynthesis, altered DA release and defects in the tryptophan-derived serotonin (5-HT) and the serotonin receptor, transporter and synaptic vesicle trafficking systems have been implicated in several of the longest held pathoetiologic- and pathogenic-hypotheses for schizophrenia (Zhang et al., 2014; Baou et al., 2016; Cocchi et al., 2016; Egbujo et al., 2016; Garay et al., 2016; Howes et al., 2017). The overlapping participation of dopaminergic and serotoninergic pathways, especially with DA-metabolizing COMT pathways and serotonin (5-HT) reuptake inhibitors (SSRIs; Zoloft, Paxil, Prozac and other antidepressants) and blockage of serotonin transporter activities are remarkable. The duality of DA and serotonin signaling is further underscored by the actions, for example, of antipsychotic medications such as Brexpiprazole (Rexulti^®^; Lundbeck, Otsuka Pharmaceutical), a partial agonist at DA D2 and serotonin 5-HT1A receptors and an antagonist at serotonin 5-HT2A receptors, recently approved by the US Food and Drug Administration (US-FDA) for the treatment of acute or advanced cases of schizophrenia (McEvoy and Citrome, 2016). Such dual-action antipsychotic medications have shown positive outcomes, such as the significant reduction of relapse in patients, in two independent Phase 3 randomized controlled clinical trials for schizophrenia, and Phase 3 trials are ongoing in patients with the aggressive behaviors associated with AD (Marder et al., 2016; McEvoy and Citrome, 2016).

Of related interest is the concept of “*retrogenesis*” as it pertains to AD and schizophrenia. Retrogenesis predicts that: (i) brain functions decline in the opposite order to which they develop in humans or during evolution; and that (ii) these anatomical regions of the brain that developed last may be the first to deteriorate with the onset and propagation of NPS illness. Put another way, the expression of genes within anatomical regions known to direct high-level brain processing functions, such as the integration of visual, auditory and other sensory information associated with intellectual abilities, behavior, memory and cognition are: (i) not only especially vulnerable to the pathoetiology and pathobiology of schizophrenia or the AD process; but (ii) are the same brain regions that display a widespread loss of cortical synaptic connectivity driven by multiple risk genes that adversely affect synaptogenesis and synaptic integrity resulting in aggressive behaviors, such as those periodically encountered in AD (Ashford and Bayley, 2013; Douaud et al., 2014; Coyle et al., 2016).

## Conclusions and Summary

Just as lethargy, inactivity and passivity, and agitation and hyperactivity lie at opposite ends of the NPS spectrum, apathy and aggression lie at opposite ends of NPS behaviors associated with AD. Certain NPSs are central features of both AD and schizophrenia. Once thought to emerge primarily in the later stages of these diseases, NPSs are currently understood to manifest commonly in early disease stages and in prodromal phases such as MCI. Aggression is a common NPS in both MCI and AD, exhibits variability and fluctuation in its presentation during the course of AD, is considerably more prominent in the later stages of AD, is often associated with psychosocial stressors, may be a response to perceived or imagined threats, and requires dutiful intervention when it causes the AD patient to be a threat to their family or care-givers’ safety and well-being. Although aggressive behaviors in AD have received considerable research attention especially over the last 20 years, the molecular-genetic mechanisms involved in both functional and pathological aggression remain incompletely understood and vast gaps in our knowledge remain (Xing et al., 2012; Gallagher and Herrmann, 2015; Smagin et al., 2015). On the other hand, progress is being made on the appreciation that the regulation of complex behaviors such as aggression is controlled by an increasingly defined broad spectrum of highly interactive brain abundant neurotrophins and/or neurotransmitters—these same signaling molecules appear to have both triggering and preventing effects on aggressive behaviors. Similarly, anti-aggression medications: (i) have displayed hypervariable effects on aggressive behaviors in humans and Tg-AD models depending on the medicinal dose, age, gender and disease duration; and (ii) have displayed both inhibitory and stimulating effects on aggressive behaviors, depending largely upon the primary biological receptors where they act and what brain anatomical regions are more exposed to pharmacological treatment (Coyle et al., 2016; McEvoy and Citrome, 2016). Ancillary factors that include diet and other environmental factors, such as level of education, the patient’s lifestyle, illicit drug use, ethnicity, the severity and stage of cognitive impairment and inter-current neurological disease have a considerable influence on the degree and extent of NPSs. The interplay of microbial and host genetics and other gene inducing factors are also becoming appreciated as strong contributors to brain gene signaling that strongly impact neurological health and the age-related development of CNS disease (Zhao and Lukiw, 2015).

It is further interesting that the genetic signals associated with AD and aggressive behavior involving the AR, BDNF, COMT, DBH, NOS1 and TPH are all complex multifunctional neurotrophic factors and/or neurotransmitters: (i) that are evolutionarily ancient; and (ii) that can be highly interactive in amplifying or reducing their role in aggressive behaviors from both neuropsychological and interdisciplinary perspectives (Narvaes and Martins de Almeida, 2014; McClam et al., 2015; Coyle et al., 2016). Other factors that can impact the regulation of gene expression including diurnal regulation, oscillatory aspects, stochastic fluctuations of gene expression and their variable transcriptional regulatory roles add further layers of complexity to our understanding of the role of genetics in the aggression associated with AD. It has recently become apparent that biological mechanisms which drive feedforward loops in NPS-relevant gene expression may have strong influence on attenuating the stochasticity of brain gene expression patterns (Barger, 2016; Shearer et al., 2016). The most recent data on the genetics of aggression in AD demonstrate: (i) that both the timescale of selective gene expression; and (ii) fluctuations in the expression of those genes implicated substantially affect the function and performance of biochemical networks, and these factors further influence gene expression plasticity, especially in the transcription-enriched environment of human neurons (Figure 1; Barger, 2016; Shearer et al., 2016). It is further interesting that this work is a prime example of a remarkable congruency between GWAS-based and DNA-array and transcriptomic-array-based findings which both show similar fluctuations in the expression of gene families important in both aggression and AD. With certainty: (i) the application of novel comparative gene expression approaches that include observations of temporal changes in the activities of neurotrophic factors and/or neurotransmitters merged with imaging technologies are certain to provide an enduring foundation for the elucidation of what signaling genes in the brain are crucial contributors to complex psychiatric symptoms such as aggression; and (ii) the expression of those NPS-related genes still represent a genuinely perplexing aspect of altered behaviors associated with the AD process. The path to scientific discovery invariably originates from an awareness of what is unknown. What we do know for certain is that the genetics of AD and aggression are characterized by prominent intrinsic variabilities in the highly interactive expression of genes that are complex, dynamic and evolving, and this knowledge is sure to help guide and shape the future direction of both neurotherapeutics and NPS research.

## Author Contributions

WJL and the late James M. Hill collected and categorized all brain sample extracts, performed all experiments and wrote the article; EIR performed statistical and bioinformatics analysis.

## Conflict of Interest Statement

The authors declare that the research was conducted in the absence of any commercial or financial relationships that could be construed as a potential conflict of interest.

## Footnotes

^1^ http://www.alz.org/facts/?utm_source=google&utm_medium=search&utm_campaign=google-grants&set.custom.wt=google-grants&gclid=CIjO58yy79ICFUm2wAodlBIGmQ
