# Supplementary material for: Genetics of Aggression in Alzheimer’s Disease (AD)
Source: Front Aging Neurosci. 2017 Apr 10;9:87. doi: 10.3389/fnagi.2017.00087 (PMC5385328; doi:10.3389/fnagi.2017.00087)
Supplement: Supplementary file 1 [file Table_1.pdf]

Supplementary Table S1

	1	2	3	4	5	6	7	8	9	10	11	12	∑ sum	x mean
AR	-1.2	-2	-3	-0.9	-0.3	-2.4	0.4	-1.8	-1.5	-3.2	0	-2.6	-18.5	-1.5
BDNF	-1.8	-1	-0.05	-2.8	-3.3	-2.3	0.66	-0.5	-1.5	-3.1	-3.6	-0.6	-19.89	-1.7
COMT	-0.9	0.9	3	3.9	5.9	2.35	6.3	4.5	0.18	8.3	-1.8	1.4	34.03	2.9
DBH	-3.5	-4.3	-2.7	-2	-0.3	0.55	-4.7	1.5	-2.2	-3	-1.5	-5.4	-27.55	-2.3
NOS1	1.8	2.2	0.3	-0.45	7.8	1.3	3.2	4.6	-2.3	4	5.2	6.4	34.05	2.8
TPH1	-0.4	-0.05	-1.1	1	-1.6	1.45	0.7	0.6	-2.3	-2	2.1	1.8	0.2	0.02

Table S1 – data for 12 individual AD cases in relation to their own controls are numbered in the top row; genes of interest and common to both AD and aggression are in the left-most column; other genes may certainly be involved but were not studied in the current analysis; ∑sum is the arithmetic sum of all 12 data points; x mean is the mean of all 12 data points; all data points were significant relative to their individual controls ($p<0.05$, ANOVA); a positive value indicates up-regulation; a negative value indicates down-regulation; data derived in part using Silicon Genetics algorithms (Agilent Technologies, Redwood City CA, USA).
